# Development of a short-form Chinese health literacy scale for low salt consumption (CHLSalt-22) and its validation among hypertensive patients

**DOI:** 10.1186/s40795-022-00594-9

**Published:** 2022-09-12

**Authors:** Yanli Zhang, Hanjing Zhang, Song Li, Yuetong Li, Cunjie Hu, Hongyu Li

**Affiliations:** School of Nursing, Jinzhou Medical University, Jinzhou, 121000 China

**Keywords:** Hypertensive patients, Low salt, Health literacy, Scale, Revision and application

## Abstract

**Background:**

With the accelerated pace of people’s life and the changing dietary patterns, the number of chronic diseases is increasing and occurring at a younger age in today’s society. The speedily rising hypertensive patients have become one of the main risk factors for chronic diseases. People should focus on health literacy related to salt consumption and reach a better quality of life. Currently, there is a lack of local assessment tools for low salt consumption in mainland China.

**Objective:**

To develop a short-form version of the Chinese Health Literacy Scale For Low Salt Consumption instrument for use in mainland China.

**Methods:**

A cross-sectional design was conducted on a sample of 1472 people in Liaoxi, China. Participants completed a sociodemographic questionnaire, the Chinese version of the CHLSalt-22, the measuring change in restriction of salt (sodium) in the diet in hypertensives (MCRSDH-SUST), the Brief Illness Perception Questionnaire (BIPQ), and the Benefit-Finding Scales (BFS) to test the hypothesis. Exploratory factor analysis and confirmatory factor analyses were performed to examine the underlying factor structure of the CHLSalt-22. One month later, 37 patients who participated in the first test were recruited to evaluate the test-retest reliability.

**Results:**

The CHLSalt-22 demonstrated adequate internal consistency, good test-retest reliability, satisfactory construct validity, convergent validity and discriminant validity. The CHLSalt-22 count scores were correlated with age, sex, body mass index (BMI), education level, income, occupation, the Measuring Change in Restriction of Salt (sodium) in Diet in Hypertensives (MCRSDH-SUST), the Brief Illness Perception Questionnaire (BIPQ), and the Benefit-Finding Scales (BFS).

**Conclusion:**

The results indicate that the Chinese Health Literacy Scale For Low Salt Consumption (CHLSalt-22) version has good reliability and validity and can be considered a tool to assess health literacy related to salt consumption in health screenings.

## Introduction

The Seventh National Population Census in China showed that the population aged 65 years and above accounted for 13.50%, with an increase of 5.44% from 2010 [1]. The increasing number and more extensive base of the elderly have led to a growing prevalence of chronic diseases, such as hypertension. According to the standard blood pressure values established by the Chinese Hypertension Association in 2021, the standard blood pressure values were categorized as 130/85. By 2020, the number of people with hypertension had reached 270 million in China, and only 14.5% of these cases were well-controlled [2].

The World Health Organization (WHO) has developed several measures to address the increasing number of people diagnosed with hypertension [3, 4]. Among the affected factors, the Dietary Approach to Stop Hypertension (DASH) might be the most effective intervention for lowering BP in adults with prehypertension to establish hypertension [5, 6]. Indeed, the use of “low-salt” (i.e., 0.3 g/100 g) bread played a key role for reducting in dietary salt intake [7]. In Organization for Economic Co-operation and Development (OECD) member countries, the measure of salt reduction could be cost-effective in the prevention of hypertension [8]. In India, reduced-sodium added-potassium salt significantly reduced SBP in patients with hypertension [9]. According to previous studies, dietary modifications for lowered BP include reduced sodium intake, weight loss, moderation of alcohol consumption, and healthy dietary patterns [10, 11]. Cross-sectional studies have shown that salt intake is significantly associated with hypertension [12, 13], and sodium intake is a potential factor independent of other influencing factors [14]. In conclusion, a low-salt diet has been promoted worldwide, which shows that it is necessary to improve abnormal eating behaviors and habits in a person’s dietary lifestyle [15].

A study has shown that assessing people’s health literacy with cardiovascular health problems will help develop appropriate health strategies to prevent associated complications [16]. However, consumer understanding of salt and salt reduction is still limited in China [17]. The COVID-19 epidemic may have influenced the salt-related knowledge and behaviors of hypertensive patients in China [18]. According to the China National Nutrition and Health Survey results, the average daily salt intake was 9.3 g per person. Although there was a decrease of 1.2 g compared to the results published in 2015, it still much higher than the maximum daily salt intake (6 g/d) recommended by “Dietary Guidelines for Chinese Residents” [19]. The World Health Organization’s (WHO) Global Action Plan for the Prevention and Control of Noncommunicable Diseases (2013–2020) said that salt intake should be correspondingly reduced by 30% [20]. The study of China’s Zhejiang Province is a population-based survey of 7512 residents. Only 12.0% of participants once used or were currently using Salt-Restriction Spoons (SRS) [21]. One study showed that school-based health education levels significantly reduced systolic BP among parents [22]. However, in another study, the effects of sodium reduction were more evident at higher starting blood pressure levels, older age, and among non-white populations [23]. It shows that reducing salt intake and realizing low salt intake health literacy is essential for a person’s health. Therefore, it is especially significant for Chinese people to know about the health literacy of low salt intake and to control the amount of salt consumed in daily life.

From the available studies, it is known that several scholars have developed tools for assessing various aspects of salt. Based on an extension of the Theory of Planned Behavior (TPB), Cornelio (2009) developed an instrument used to study the determinant factors of salt consumption among hypertensive subjects. The final tool, comprises three different behaviors related to salt consumption [24]. Roghayeh Chenary developed an instrument based on the same TPB to measure the factors affecting salt-restriction behaviors among women. There are three different salt intake behaviors, including adding salt during cooking, at the table, and purchasing salty food [25]. The dietary sodium restriction questionnaire (DSRQ) of 15-item is composed of three subscales: attitude, subjective norm, and perceived behavioral control [26]. The Scored Sodium Questionnaire (SSQ) is used in the routine clinical care of patients with chronic kidney disease (CKD) [27]. The Dietary Sodium Reduction Self-Care Agency Scale (DSR-SCA Scale) of 24 items measures the capability or self-care agency of lowering salt consumption in older adults with hypertension [28].

According to the literature, there are many studies on the influence of salt intake (sodium) on hypertension and other chronic diseases. However, there are not only a few surveys about knowledge, attitudes, and dietary practices related to low salt consumption but also a validated scale on health literacy related to low salt consumption in mainland China. Therefore, this study conducted cultural adjustment of CHLSalt-HK and applied it to the mainland Chinese population to adapt the instrument and evaluate its validity and reliability. Furthermore, we provide a theoretical basis and data support for healthy quality and low salt.

## Methods

### Design and participants

A cross-sectional survey was conducted in Liaoning Province, China, from August to December 2021. The participants were hypertensive patients from Fuxin, Chaoyang, Panjin and Jinzhou. All patients provided informed consent before participating in the study. The research procedures complied with the ethical standards of the Ethics Committee of Jinzhou Medical College, the 1964 Helsinki Declaration, and its later amendments. A total of 1577 hypertensive patients were recruited for the survey. During the survey, the authors and investigators explained the study’s purpose and methods to patients. The questionnaires were individually delivered to each participant and completed in the presence of the authors and the investigator. The participants were encouraged to give truthful answers. Subjects who had not fully completed the scale and provided questionnaires with obvious logical errors were excluded. The remaining 1472 patients (93.34%) were retained. The survey was anonymous, except that 37 patients in Jinzhou were required to write their names as the test-retest participants. One month later, 37 patients who participated in the first test were recruited to evaluate the test-retest reliability.

### Revision process

#### The first step

We obtained permission from Dr. PH Chau to revise and verify the Chinese Health Literacy Scale For Low Salt Consumption. The Chinese Health Literacy Scale For Low Salt Consumption was formulated in 2015 [29].

#### The second step

We then compared the questions with the corresponding culture of the researchers, companies (Hanjing Zhang, Song Li, et al.)and professors (Hongyu Li, PH Chau, et al.). We revised some items to conform to the diet. Considering the different food preferences between Hong Kong and mainland China, we replaced some food examples with this scale. Based on the literature review, the team and experts removed some items based on evidence, considering that some items were outdated and unsuitable for future applications in various countries. When filling in the questionnaire, the patients felt that their functional literacy and knowledge of international standards were similar. After experts and a literature review, the two dimensions were combined. Regarding statistics, experts said a scale should have no more than seven dimensions. Finally, a pilot study was conducted among 37 patients with hypertension. They were invited to complete the scale and then were asked about their understanding of the scale’s introduction section, items, and options. We communicated with the survey respondents, who reported that they had no difficulty in understanding the content of each scale item, and the revised scale was obtained.

#### The third step

The revised scale was investigated in 469 older people referred 60 years old and older.. Through the statistical analysis, the revised scale indicated well reliability and validity. So, we ended up with the Chinese Health Literacy Scale For Low Salt Consumption of 22 items. The scale was tested on 300 non-hypertensive subjects to determine its explanatory degree and stability. ROC curve analysis was conducted with hypertensive group as experimental group and non-hypertensive group as control group. The objective of the ROC curve is to understand the trend and critical value of hypertensive low-salt health literacy.

#### The fourth step

We applied the CHLSalt-22 scale to 1003 hypertensive participants The purpose of this step was to use the revised scale in different age groups, further verify the applicability of the scale in inland Chinese population, and reveal the low-salt health literacy of inland Chinese population.

### Measurements

All participants completed the CHLSalt-22, the measuring change in restriction of salt (sodium) in the diet in hypertensives (MCRSDH-SUST) [30], the Brief Illness Perception Questionnaire (BIPQ) [31], and the Benefit Finding Scales (BFS) [32]. Participants were also asked to complete a checklist assessing sociodemographic variables (e.g., sex, age, and income). Height and weight were also measured to calculate each participant’s body mass index (BMI) Participants were categorized as underweight (< 18.5 kg/m^2^), normal weight (18.5–23.9 kg/m^2^), overweight (24–27.9 kg/m^2^), and obese (≥28 kg/m^2^) based on Chinese criteria of weight for adults [33].

### The Chinese health literacy scale for low salt consumption (CHLSalt-22)

The CHLSalt-22 consists of 22 items with three items assessing Functional literacy, four items assessing Salty food knowledge, three items assessing Disease knowledge, three items assessing Myths about salt intake, three items assessing Salt intake attitudes, three items assessing Salty food consumption, and three items assessing Nutrition label practices. The questions’ responses were in the form of either a 5-point Likert scale or four multiple-choice options. For the Likert-scale questions, the most favorable option scored two points, the following profitable option scored one point, and the remaining three scored 0 points. The correct answer scored 2 points for multiple-choice questions, a score of 2 was assigned to the correct answer, and the remaining options scored 0 points. The total score was calculated by summing up the scores for each item [29].

### The measuring change in restriction of salt (sodium) in the diet in hypertensives (MCRSDH-SUST)

The Continuous Behavior Change Sub-scale (McRsdh-sust) includes nine items, including emotional change (three items), behavior change practice (three items) and social environment change (three items). Likert 5 rating method was used for this scale, 0 = “very uncertain”, 1 = “uncertain”, 2 = “not certain”, 3 = “certain”, 4 = “very certain”. The higher the total score, the more likely people with hypertension will change their salt-restricted diet [30].

### The brief illness perception questionnaire (BIPQ)

The Brief Illness Perception Questionnaire (BIPQ) was compiled by Broadben et al. It includes two dimensions of cognitive disease symptoms and the degree of understanding for patients with emotional disorders, with eight items as a self-assessment questionnaire. The cognitive disease representations include disease influence course, symptom recognition, individual control, and treatment control. The emotional disease representations include disease worry and mood, with each item rated from 0 to 10. 0 = “degree of tiny “,10 = “degree of extremely strong”,with a total score of 80 points [31]. The higher the score, the stronger negative perception.

### The benefit finding scale (BFS)

The Chinese scale includes 19 items, all scored on a 4-point Likert scale, assessing 1 to 4 points from none to very much. The total score was 19–76, and the higher the score was, the higher the perceived benefit level of the participant. The Cronbach’s α coefficient of the scale in this study was 0.910 [32].

### Statistical analysis of data

Data analysis was performed using SPSS 26.0 and Mplus 8.0. Given that all the items were dichotomous, Kuder-Richardson’s α (KR-20) was used to assess the internal consistency of the CHLSalt-22. The test-retest correlation coefficient (intraclass correlation coefficient, ICC) was used to calculate the scale’s stability. Values of ICC were interpreted as follows: > 0.75 was excellent, between 0.40 and 0.75 was fair to good, and < 0.40 was poor [34]. The content validity index (CVI) and Pearson’s correlation coefficients between items and total scores were used to evaluate the scale’s content validity. The CVI includes item-level content validity index (I-CVI) and average S-CVI (S-CVI/Ave) [35]. Each expert chose the relevance of each item to the corresponding dimension. A 4-point rating scale was used to calculate CVI (1 = no relevance, 2 = low relevance, 3 = strong relevance, 4 = very strong relevance).EFA and CFA were used to examine the construct validity of the CHLSalt-22. Data were divided into two groups. Sample 1 consisted of 469 hypertensive patients (53.3% women, mean BMI = 26.61, SD = 3.12), while sample 2 consisted of 1003 hypertensive patients (32.2% women, mean BMI = 26.89, SD = 3.61). The factor ability of the correlation matrix was assessed with the Kaiser–Meyer–Olkin (KMO) statistic and Bartlett’s test for sphericity [36], and EFA was conducted on sample 1. A scree plot was used to constructed to determine the number of factors. CFA was performed on Samples 1 and 2, and the test level was α = 0.05. To assess the quality of the factor model, we estimated the following indices: minimum function chi-square (χ^2^), comparative fit index (CFI), Tucker-Lewis index (TLI), standardized root mean residual (SRMR), and the root mean square of approximation (RMSEA). An acceptable model should have a χ^2^/df < 3, an RMSEA and an SRMR< 0.08 [37], and a CFI and a TLI > 0.9 [38]. To assess convergent and discriminant validity, we used the path coefficient of CFA to analyze composite reliability (CR) and average variance extract (AVE). An acceptable model should have a CR>0.7 and an AVE>0.45 [39]. Through SPSS 26.0 to analyze the predictive validity of the CHLSalt-22 scale. The area under the ROC curve was>0.70, and the best cut-off points for the CHLSalt-22 scale were analyzed. An AUC of 0.5 represents a test with no discriminating ability, while an AUC of 1.0 represents a test with perfect discrimination [40]. Independent sample t-tests or single-factor ANOVA of the difference in the total score of symptom counts between sociodemographic classifications and Bonferroni’s test were used to calibrate the inspection level for pairwise comparisons. The correlation between the CHLSal-22 t count score and MCRSDH-SUST, BIPQ, and BFS was evaluated by calculating Pearson’s correlation coefficient. The CHLSalt-22 count score was taken as the dependent variable, and the classified and continuous variables were used as independent variables for multivariate linear regression analysis. The multi-classified disordered variables were set as dummy variables according to the requirements of multivariate linear regression for independent variables. The significance level was set at *P* < 0.05.

## Results

### Descriptive statistics

Sociodemographic variables differed in the CHLSalt-22 score with P<0.05. Age, Education level, occupation, and income were significantly different. Other demographic characteristics of the study participants are shown in Table 1.

**Table 1:** Sample characteristics (*n* = 1472)

Characteristics	Total (*N* = 1472) N (%)/M ± SD	The CHLSalt-22 score (M ± SD)	t/F	*P*
**Sex**			**1470**	**0.802**
Man	899(61.1)	17.63 ± 7.43		
Woman	573(38.9)	17.52 ± 8.43		
**Age**			**3.821**	**0.022**
≤ 39	221(15.0)	17.75 ± 0.28		
40–59	695(47.2)	18.10 ± 0.09		
≥ 60	556(37.8)	16.88 ± 0.11		
**Education level**			**20.692**	**0.000**
Primary school and below	463(31.5)	16.16 ± 0.13		
Secondary school	467(31.7)	17.10 ± 0.13		
High school	281(19.1)	17.80 ± 0.21		
University or above	261(17.7)	20.74 ± 0.23		
**Occupation**			**28.870**	**0.000**
Self-employ	278(18.9)	17.27 ± 0.21		
Worker	623(42.3)	19.27 ± 0.10		
Farmer or other	571(38.8)	15.90 ± 0.10		
**Exercise**			**2.766**	**0.063**
no	349(23.7)	16.86 ± 0.18		
Yes,Once in a while	803(54.6)	17.62 ± 0.08		
Yes, at less than once a week	320(21.7)	18.28 ± 0.19		
**Income** (RMB)			**17.515**	**0.000**
<1000	184(12.5)	15.70 ± 0.32		
1000–1999	470(31.9)	16.07 ± 0.13		
2000–2999	208(14.1)	19.13 ± 0.29		
≥ 3000	610(41.4)	18.79 ± 0.10		
**BMI** (kg/m^2^)	26.80 ± 3.46		**–**	**–**
Underweight	7(0.5)	16.00 ± 8.7		
Normal weight	284(19.3)	18.36 ± 0.22		
Overweight	682(46.3)	18.00 ± 0.10		
Obese	499(33.9)	16.60 ± 0.12		

### Reliability analysis

The CHLSalt-22 consists of 22 items. Thirty-seven participants took part in this analysis. The KR-20 and split-half reliability of the CHLSalt-22 were 0.815 and 0.713, respectively. After 1 month, the test-retest ICC of the CHLSalt-22 was 0.982.

### Construct validity analysis

**Fig. 1:**
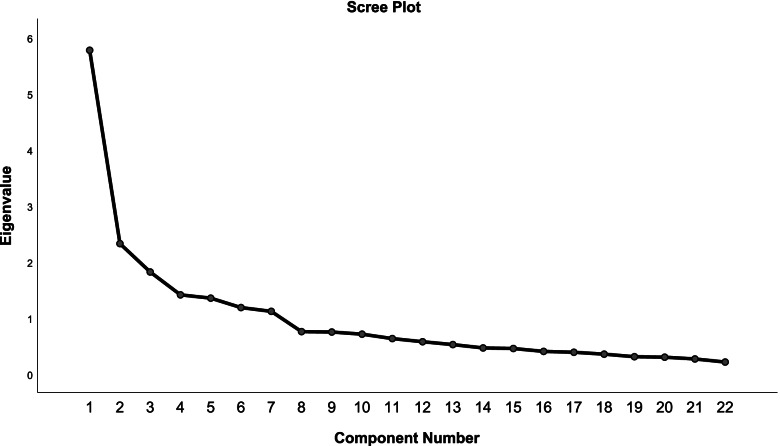
Screen plot of exploratory factor analysis for the revised version of the CHLSalt-22

The result of the Harman single-factor test analyzes Common Method Biases (CMB) was 26.228 < 40, and the contribution rate was 67.993% > 60% [41]. The statistically significant results of Bartlett’s test of sphericity (χ^2^(231) = 3815.612, *p* < 0.001) and the Kaiser-Meyer-Olkin Measure of Sampling Adequacy > 0.80 (KMO = 0.815) indicate that the data meet the conditions for using factor analysis. Therefore, the first principal component analysis (PCA) was performed to determine the likely number of factors. As a result, seven of the actors that explained a total of 49.709% of the variance had initial eigenvalues > 1 each. The scree plot further confirms the seven-factor structure. After varimax orthogonal rotation, these seven extracted factors explained 10.985, 21.925, 32.716, 42.813, 52.232, 61.396 and 67.993% of the variance, respectively. The scree plot is presented in Fig. 1.

**Fig. 2:**
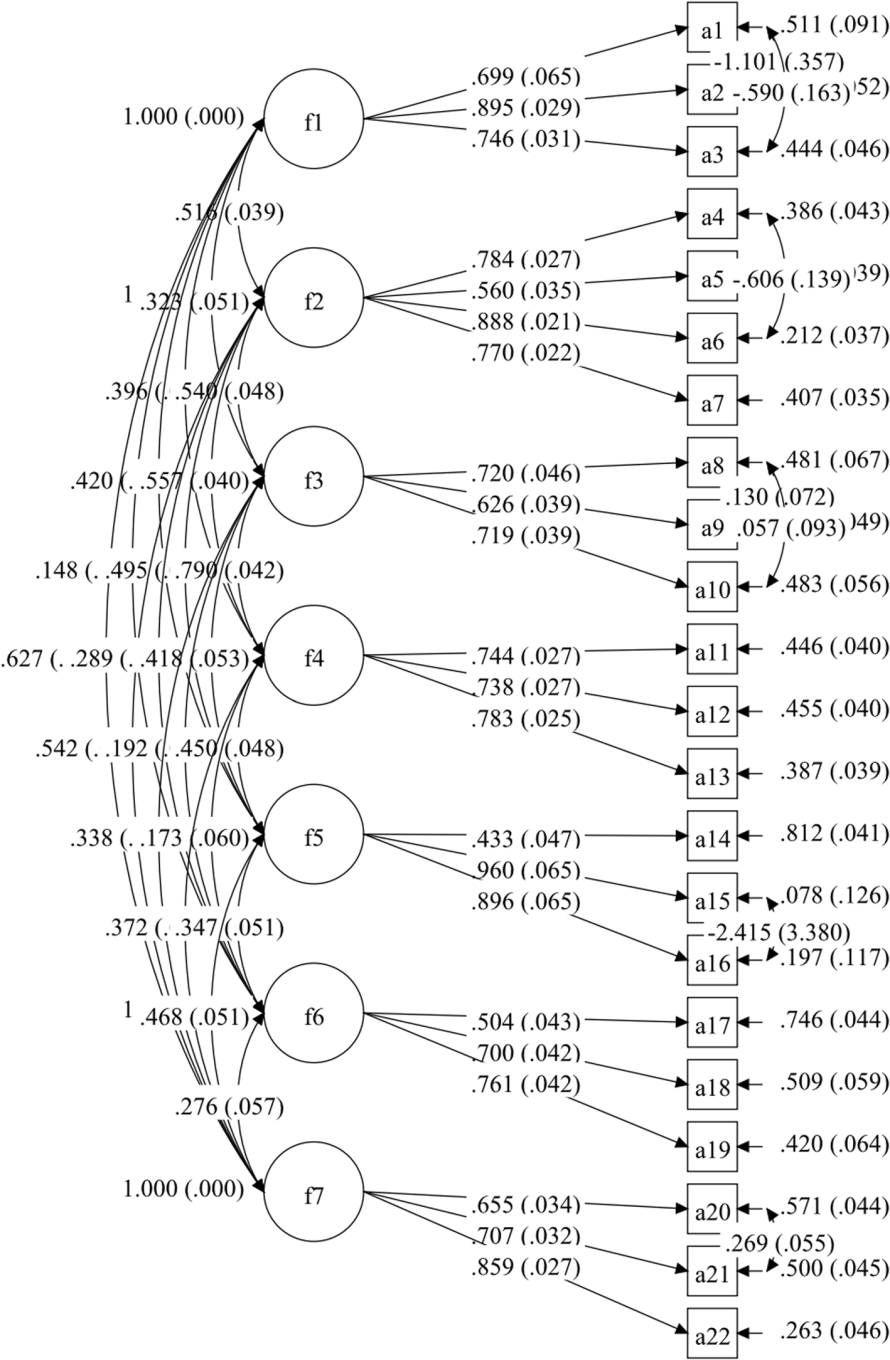
Standardized seven-factor structural model of the CHLSalt-22 (*n* = 469); F1 (Functional literacy, 3 items), F2 (Salty food knowledge, 4 items);F3 (Disease knowledge, 3 items), F4 (Myths about salt intake, 3 items);F5 (Salt intake attitudes, 3 items), F6 (Salty food consumption, 3 items);F7 (Nutrition label practices, 3 items)

Table 2 presents the factor loading of each item. All the correlation coefficients were more prominent than 0.40 and statistically significant at *P* < 0.01. A CFA was performed on Sample 1 (*n* = 469). This study evaluated the two-scale models, and the results showed the fitting index of the seven-factor model (Table 3). The structural equation model and the standardized regression coefficients of the seven-factor model of the CHLSalt-22 are shown in Fig. 2.

**Table 2:** Factor loadings of the CHLSalt-22 (*n* = 469; salient factor loadings are indicated in italics)

Item	Estimate
1. Which statements best describe the relationship between salt and sodium?	*0.699*
2. What is the daily limit of salt intake (in grams) recommended by the World Health Organization for an adult?	*0.895*
3. Which type of biscuits would you choose if you wish to minimize salt intake?	*0.746*
4. How much is the salt content of Lunch meat(100 g)?	*0.784*
5. How much is the salt content of Instant noodles with seasoning powder(100 g)?	*0.560*
6. How much is the salt content of Ketchup or Salad dressing (100 g)?	*0.888*
7. How much is the salt content of Oyster sauce(100 g)?	*0.770*
8. Do you agree that high blood pressure can be caused by high salt intake?	*0.720*
9. Do you agree that cardiovascular disease can be caused by high salt intake?	*0.626*
10. Do you agree that diabetes mellitus can be caused by high salt intake?	*0.719*
11. Sodium intake can be reduced by replacing salt with plenty of Chicken essence during cooking.	*0.744*
12. Most foods available at restaurants(e.g,Chinese restaurants,fast food restaurants) are high in salt.	*0.738*
13. Drinking more water can neutralize salt intake from my diet.	*0.783*
14. Most low salt foods taste bad.	*0.433*
15. I feel too much pressure to eat a healthy diet.	*0.960*
16. Limiting the amount of salt intake is essential to my health.	*0.896*
17. Add salt or sauce, or condiments to the table.	*0.504*
18. Consume canned foods.	*0.700*
19. Consume salted fish, salted vegetables, and salted duck eggs.	*0.761*
20. Pay attention to whether the food is labeled as “No added salt” or “Low in salt”.	*0.655*
21. Read the sodium content stated on the food package nutrition labels.	*0.707*
22. Purchase foods according to the sodium content on the nutrition labels.	*0.859*

**Table 3:** Confirmatory factor analysis of the CHLSalt-22 with different models

Model	χ^2^	df	χ^2^/df	CFI	TLI	SRMR	RMSEA[90%CI]
1	354.170	181	1.957	0.956	0.944	0.041	0.045[0.038–0.052]
2	632.060	182	3.473	0.922	0.901	0.049	0.050[0.045–0.054]

*χ*^*2*^ Chi-square, *df* Degrees of freedom, *CFI* Comparative fit index, *TLI* Tucker-Lewis index, *SRMR* Standardized root mean residual, *RMSEA* Root mean square error of approximation, *90% CI* 90% confidence interval, *M1* 469 samples structure model, *M2* 1003 samples model

### Convergent validity and discriminant validity

The path coefficient of CFA was to analyze the composite reliability (CR) and average variance extract (AVE). The critical ratios among the seven common factors in the CHLSalt-22 were over 0.7, and all of the average variance extraction values were over 0.45. The convergent and discriminant validity of the CHLSalt-22 is shown in Table 4.

**Table 4:** Convergent validity and discriminant validity of the CHLSalt-22

Factor	CR	AVE	F1	F2	F3	F4	F5	F6	F7
F1	0.826	0.615	**0.784**						
F2	0.842	0.577	0.516^**^	**0.760**					
F3	0.731	0.476	0.323^**^	0.540^**^	**0.690**				
F4	0.799	0.570	0.396^**^	0.557^**^	0.790^**^	**0.755**			
F5	0.828	0.637	0.420^**^	0.495^**^	0.418^**^	0.450^**^	**0.798**		
F6	0.700	0.441	0.148^**^	0.289^**^	0.192^**^	0.173^**^	0.347^**^	**0.664**	
F7	0.787	0.556	0.627^**^	0.542^**^	0.338^**^	0.372^**^	0.468^**^	0.276^**^	**0.746**

*CR* Composite reliability, *AVE* Average variance extract; **: *P* < 0.01; *: *P* < 0.05

The bold numbers on the diagonal lines of the table are the square root of the extraction amount of the mean-variance of the corresponding dimensions, and the non-diagonal numbers are the inter-dimensional correlation coefficients

### Content validity

The expert group comprises seven experts: two dieticians, three nursing experts proficient in Chinese and English, and two psychiatrists. The content validity analysis result shows that the I-CVI of the CHLSalt-22 scale is 0.857–1, and the S-CVI /Ave is 0.968.

### Prediction validity

**Fig. 3:**
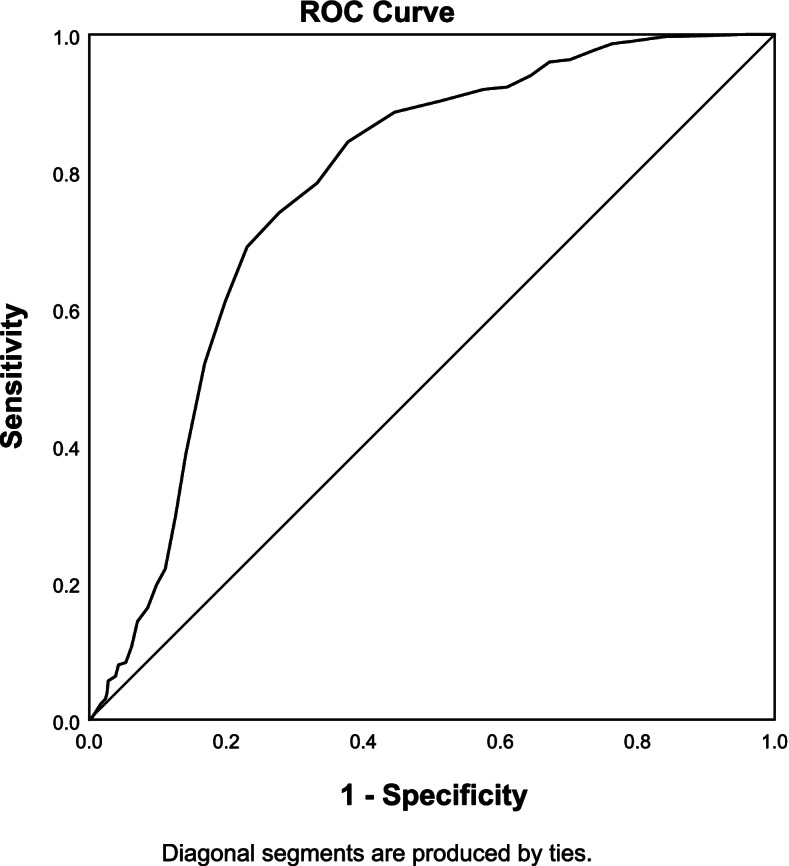
The ROC curve of the CHLSalt-22

The ROC curve of the CHlsalt-22 was drawn in this study, with hypertension as a reference. According to the Youden’s Index (YI) maximization principle [42, 43], 16.5>17 was the best cut-off value for the CHLSalt-22 scale. The area under the ROC curve for the scale was 0.774 (p<0.01), with a sensitivity of 0.843, specificity of 0.623, and 95%CI (0.741–0.807). The ROC curve for the CHLSalt-22 is shown in Fig. 3.

### Correlation and influencing factors analysis

Table 5 presents the factors associated with the CHLSalt-22 score: the CHLSalt-22 score was positively correlated with the BIPQ, BFS, and MCRSDH-SUST. The effects of different sociodemographic groups on the CHLSalt-22 score were assessed using bilinear regression. Sociodemographic groups were considered categorical predictors (with one category as the reference group), and the CHLSalt-22 score represented the continuous outcome variable. The results of the multivariate regression analysis results are shown in Table 6.

**Table 5:** Pearson’s correlations between the CHLSalt-22 count and MCRSDH-SUST,BIPQ and BFS

	1	2	3	4
1.CHLSalt-22	–			
2.MCRSDH-SUST	0.437^**^			
3.BIPQ	0.226^**^	0.484^**^		
4.BFS	0.348^**^	0.513^**^	0.340^**^	–

*CHLSalt-22* Revised Chinese Health Literacy Scale For Low Salt Consumption, *BIPQ* The Brief Illness Perception Questionnaire, *BFS* Benefit Finding Scales, *MCRSDH-SUST* The Continuous Behavior Change Sub-scale; ** *p* < 0.01; * *p* < 0.05

**Table 6:** Results of multiple linear regression models of factors influencing the CHLSalt-22 scores of subjects with different characteristics

Variate	β	SD	Β’	t	*P*	R^2^	DR^2^	F	*P*
Model 1						0.074	0.070	16.799	0.001
Sex	1.307	0.452	0.081	2.889	**0.004**				
Age	1.330	0.576	0.084	2.307	**0.021**				
BMI	−0.195	0.058	−0.086	−3.349	**0.001**				
Income	2.317	0.630	0.103	3.676	**0.000**				
education level	2.574	0.566	0.126	4.550	**0.000**				
Occupation	1.461	0.493	0.092	2.966	**0.003**				
Model 2						0.212	0.211	134.649	0.001
MCRSDH-SUST	0.412	0.038	0.351	10.737	**0.000**				
BFS	0.116	0.022	0.168	5.145	**0.000**				

*BMI* Body mass index, *BFS* Benefit Finding Scales, *MCRSDH-SUST* The Continuous Behavior Change Sub-scale, *P P*-Value, Bold values correspond to statistically significant correlations (*p* < 0.05)

### Health literacy related to salt consumption and sociodemographic differences

The CHLSalt-22-diagnosed health literacy related to salt consumption count was 17 in patients with hypertension (SD = 17.58; range = 0–44). According to the ROC of the CHLSalt-22 analysis, the higher the low salt health literacy score, the more likely they are to have high blood pressure. It proves that people with hypertension are aware of the prevention of hypertension and other diseases and pay special attention to the salt content of diet in their daily lives. Many studies have shown that people with high blood pressure have better health literacy [32]. There were no significant differences in the CHLSalt-22 between men and women or between different exercise frequencies. However, there were statistically significant differences in age, education level, occupation, income, and BMI. Table 1 presents the results. There are differences in the level of health literacy, which is similar to the results of previous studies conducted at home and abroad [44, 45]. Many studies have shown that age, sex, and education level are all associated with health literacy in older adults [46–48]. However, a part of the result of the original scale was different [29]. This result is due to various cultures, customs, and demographic characteristics.

Sex, age, BMI, income, education level, occupation, BFS, and MCRSDH-SUST were the influencing factors of the CHLSalt-22. It reflects that health literacy is influenced by individual and environmental social determinants [49]. Nevertheless, BMI was negatively correlated with the CHLSalt-22 and other characteristics were positively correlated with the CHLSalt-22. These results suggest that the higher weight of hypertensive patients, the lower the health literacy of low salt intake, and the less attention to salt intake in the normal diet process. The CHLSalt-22 count score increased by 2.317 points for each one-unit increase in income, by 2.574 points for each one-unit increase in education level, by 1.461 points for each one-unit increase in occupation, and decreased by 0.195 points for each one-unit increase in BMI. This trend is consistent with the results of previous studies [50–53]. Through the descriptive statistical analysis, this study showed that different levels of education and monthly income have different impacts on each of the seven dimensions. Different income levels showed the same trend in the seven dimensions: the people with higher income have higher mean scores in each dimension. The effects of low to high education levels on Functional literacy (1.83, 2.48, 2.65, 2.48), Salt intake attitudes (2.08, 2.52, 2.36, 3.42), and Nutrition labeling practices (4.19, 4.25, 4.23, 4.42) showed a wave-like pattern of increase in the three dimensions. Although there are different changes in the mean values of different educational levels in the seven dimensions, the overall change direction is positive. It indicated that the CHLSalt-22 scores helped assess health literacy related to salt consumption in health screenings and provided practical guidance for salt reduction in subjects with low health literacy.

## Discussion

The CHLSalt-HK is the first validated scale for assessing health literacy related to low salt intake among elderly Chinese adults in Hong Kong. The 49-item scale has a possible score ranging from 0 to 98, with a higher score indicating higher health literacy related to low salt intake. However, the CHLSalt-22 scale has possible scores ranging from 0 to 44. Our sample of Chinese adults had a mean score of 17.58 and a standard deviation of 7.83. The median score was 18.00, the observed minimum score was 2, and the observed maximum score was 41. None of the respondents scored a maximum of 44 or a minimum of 0; therefore, floor or ceiling effects were unlikely to occur. The scale previously studied by the doctor is applied to the elderly. Considering that the population of hypertensive patients is getting younger and younger, this scale was used for the adult population of any age [29].

This study shows that the CHLSalt-22 has a seven-factor structural solution and has good authentic characteristics. This study’s reliability analysis showed that the scale’s internal consistency coefficient met the statistical requirements, and the CHLSalt-22 test-retest reliability was good, indicating that the scale has good stability over time [34, 54]. Furthermore, the CHLSalt-22 had good construct validity, convergent validity, discriminant validity [39, 55], content validity [56], and prediction validity [43]. A seven-factor structure of the CHLSalt-22 was confirmed as the best solution for the scale through CFA, which differs from the theoretical conception of the original scale [29]. The items were removed from 49 to 22, and the factors ranged from 8 to 7. First, during the revision process, items that did not conform to mainland Chinese eating habits were adjusted and cross-culturally adjusted, which affected the original structure to a certain extent. Second, this difference may be related to the sample population and region. The survey samples were mainly from northeast China. Therefore, people from different areas may have different subjective experiences of salt consumption and an understanding of the concept of salt consumption. Other possible reasons were their different lifestyles, eating habits, and cultures. China has a vast territory with different dietary compositions. Our samples were mainly from Northeast China, where the diet is high in salt [57]. A retrospective study of significant changes in the dietary structure of Chinese adults showed that consumption of cooking oil and salt was substantively far above the recommendations [58]. Dietary differences in the sample population may be a fundamental reason for the different results. Finally, we measured varying levels of people, shortened the time for people to fill in the questionnaire, and ensured the authenticity of the answers. Repetitive or complex questions were removed to make the scale more universal. In this process, the professor’s consent was obtained. From a content point of view, there are relatively reasonable explanations for various factors.

Based on the construct validity analysis, twenty-seven items were removed. From a content point of view, there are relatively reasonable explanations for the various factors. According to the previous studies [21, 59–61], the salt restriction policy was promoted in many countries, including China, and some people used a 2-g salt-restriction spoon for cooking. Therefore, we think the item (how many grams of salt does one teaspoon of salt have?) should be removed. Item 3 is similar to the item (refer to the following nutrition labels of various canned soups, which of the canned soups has the highest salt content?). They all examined the relevant knowledge of nutrition labels, and we think it is suitable for such a question. According to literature, high salt levels affect many diseases [8, 62, 63]. However, in recent years, the highest mortality rates in China have been for cerebrovascular disease, hypertension, and diabetes [64]. So, we only leftover these three items (8,9,10). The 16th item and the item (I worry about the serious health problems that are caused by eating salty foods.). Although the expression is the opposite, the meaning is the same. Considering the actual Chinese diet and table salt is one such condition [65]. We summed up item (add salt at the table.) and item (add sauce or condiments at the table.) into one item, named the 17th item. The 17th item and the item (only by adding salt and sauces while cooking can the taste of food be enhanced.) both refer to the act of adding salt and sauces during cooking. Item 17 is opposite to the one for minimizing my salt intake, and the reverse of the assignment proves the same. The 17th, 18th, and 19th items are similar to the item (I enjoy eating salty foods.). The 20th, the 21st, and 22nd items are similar to the item (I am concerned about the salt content in foods.) and the item (I am confident that I can control my daily salt intake.). The item (only by adding salt and sauces while cooking can enhance the taste of food be enhanced.), the item (I enjoy eating salty foods.), the item (I enjoy eating salty foods.), and the item (I am confident that I can control my daily salt intake.) were considered to be emotionally charged. There is a possibility of misinterpretation or even deviation in the investigation. The items (consume fast food, potato chips, bread, instant oatmeal, sliced cheese and hamburger) do not belong to the diet of mainland Chinese. During the investigation, respondents realized they owned different salts through disparate cooking. For people in northern China, BBQ is a typical and intangible cultural heritage food that is prone to deviation during its investigation. Due to regional differences, many rural residents or other people do not understand salted snacks and preserved fruits well and have deviations in measurement. The revised version of the CHLSalt-22 also contains the three most common domains of health literacy identified by Frisch et al.: functional literacy, factual and procedural knowledge, and awareness [66].

Based on the cut of values of BMI for Chinese adults [33], there were 7 (0.5%) underweight participants, 284 (19.3%) normal weight participants, 682 (46.3%) overweight participants, and 499 (33.9%) obese participants. In this study, BMI showed a small, passive association with the CHLSalt-22 scores (*rs* = − 0.118, *P*<0.001). Several studies have reported similar findings. For example, in a survey of Olyani S, the negative correlation between the female adolescent students’ health literacy count scores and BMI was − 0.233 [67]. Additionally, a previous survey using the Nutrition Literacy Assessment Instrument scale showed that BMI was associated with nutrition literacy and dietary habits [68]. Compared with people in the Risk Assessment and Management Program (RAMP) in a cluster-randomized survey, BMI showed no significant changes and a significantly higher BP control rate [69]. Obesity is often associated with salt-sensitive hypertension [70]. In addition, for children and students, the level of health literacy of their parents and themselves also play a crucial role in their weight [71–74]. Nutritional literacy is vital in preventing, predicting, and treating certain chronic diseases [68, 75]. It was confirmed that more in a low-fat diet and higher health-related quality of life and health literacy [46]. Given the complexity of these findings, more research is needed to identify interventions for health literacy and elucidate the associations between BMI, BP, and weight.

We found a positive correlation among the CHLSalt-22 score, BIPQ, BFS and MCRSDH-SUST. With every increase of one SD in the BFS, the CHLSalt-22 score increased by 0.168 SD. 0.351 SD increased the score increased by the CHLSalt-22 for each increase of one SD in the MCRSDH-SUST. MCRSDH-SUST is defined as changes in salt restriction dietary behavior in hypertensive patients, and it may affect the quality of life and health [30]. A previous study reported inconsistent cognition and behavior in patients on a salt-restricted diet. Although patients met the requirements of a salt-restricted diet, some patients did not implement it [76]. Few studies have examined the relationship between low salt health literacy and disease perception and benefit discovery. Still, many researchers have studied the changes of health literacy in behavior and cognition [77, 78].

Several limitations of this study should be considered when interpreting its findings. First, the sample was conveniently obtained. Our sample had a high proportion of male participants, which may limit the generalizability of our results to other populations. Further investigation into the angle of sex variation could offer valuable insights. Second, this study focuses on salt health literacy and provides a good assessment tool for future salt knowledge and health worldwide. Therefore, future studies should assess the reliability and validity of the CHLSalt-22 in other countries and evaluate the level and characteristics of health literacy related to salt consumption among different samples. Third, this study did not thoroughly explore other fields related to the scale, such as intervention research. In the future, we will explore more aspects to enrich the content of salt health literacy and provide a theoretical basis for achieving health for all.

## Conclusion

The CHLSalt-22 scale, which supports a seven-factor structure, is reliable. Therefore, it can be used as a short method of assessing health literacy in low-salt screening. Health literacy of low salt is associated with sex, age, BMI, income, education level, and occupation. Future research should examine the psychometric properties of the revised CHLSalt-22 scale across different locations in China. In addition, the potential predictors of low salt intake in hypertension should be further determined. We think measurement is only the first step, and how to change behavior is the next step in future research.

## Data Availability

The data set is available from the corresponding author upon reasonable request.

## Abbreviations

CHLSalt-22: A short-form Chinese Health Literacy Scale For Low Salt Consumption
MCRSDH-SUST: The Measuring Change in Restriction of Salt (sodium) in Diet in Hypertensives
BIPQ: The Brief Illness Perception Questionnaire
BFS: The Benefit-Finding Scales
WHO: World Health Organization
DASH: Dietary Approach to Stop Hypertension
OECD: Organization for Economic Co-operation and Development
SRS: Salt-Restriction Spoons
TPB: Theory of Planned Behavior
CKD: Nchronic kidney disease
KR-20: Kuder-Richardson’s α
ICC: Intraclass correlation coefficient
CVI: Content validity index
TLI: Tucker-Lewis index
CR: Composite reliability
AVE: Average variance extract
YI: Youden’s Index
BMI: Body Mass Index
PCA: Principal components analysis
ICC: Intraclass correlation coefcient
